# Tumour–Stroma Ratio and Platinum Resistance in Epithelial Ovarian Cancer: An Exploratory Analysis

**DOI:** 10.3390/cancers18172876

**Published:** 2026-09-05

**Authors:** Gürkan Gül, Özlem Kutlu, Duygu Ayaz, Damla Günenç, Özlem Özdemir, Celal Akdemir, Muzaffer Sancı

**Affiliations:** 1 Department of Medical Oncology, Izmir City Hospital, Izmir 35540, Turkey; ozlemacikerkutlu@gmail.com (Ö.K.); d.gunenc@yahoo.com.tr (D.G.); ozdemirozlem.md@gmail.com (Ö.Ö.); 2 Department of Pathology, Izmir Tepecik Training and Research Hospital, Izmir 35180, Turkey; ayazduygu@yahoo.com; 3 Department of Gynecologic Oncology Surgery, Izmir City Hospital, Izmir 35540, Turkey; akdemircelal@gmail.com (C.A.); drsanci@yahoo.com (M.S.)

**Keywords:** tumour–stroma ratio, epithelial ovarian cancer, platinum resistance, neoadjuvant chemotherapy, interval debulking surgery, tumour microenvironment, stromal proportion

## Abstract

**Background:** Platinum resistance remains a major therapeutic challenge in epithelial ovarian cancer (EOC). The tumour–stroma ratio (TSR) has emerged as a potential histopathological marker of tumour biology, but its clinical significance in different clinical settings remains unclear. We evaluated the association between stromal proportion, assessed using the TSR methodology, platinum resistance, and survival outcomes in patients undergoing primary debulking surgery (PDS) or neoadjuvant chemotherapy followed by interval debulking surgery (NACT+IDS). **Methods:** This retrospective study included 83 patients with epithelial ovarian cancer (EOC) who underwent either primary debulking surgery (PDS) or neoadjuvant chemotherapy followed by interval debulking surgery (NACT+IDS) between 2017 and 2024. Patients were analysed separately according to treatment strategy. Stromal proportion was assessed on primary surgical specimens in the PDS cohort and on pretreatment diagnostic biopsy specimens in the NACT+IDS cohort. For this retrospective analysis, platinum resistance was operationally defined as recurrence within six months after completion of first-line platinum-based chemotherapy. Survival outcomes were analysed using the Kaplan–Meier method, and univariable binary logistic regression was performed separately within the PDS and NACT+IDS cohorts to explore the association between stromal category and platinum resistance. **Results:** Platinum resistance occurred in 25.3% of patients. In the PDS cohort, stromal category was not associated with clinicopathological characteristics, platinum resistance, disease-free survival (DFS), or overall survival (OS). In the NACT+IDS cohort, patients in the stroma-high group had a numerically higher rate of platinum resistance than those in the stroma-low group (57.9% vs. 22.2%; Fisher’s exact *p* = 0.114). Based on univariable logistic regression analysis, the stroma-high group had higher estimated odds of platinum resistance (OR 4.81, 95% CI 0.78–29.40), although this finding did not reach statistical significance (*p* = 0.090). No significant association was observed between stromal category and survival outcomes in either cohort. **Conclusions:** In the NACT+IDS cohort, patients in the stroma-high group had a numerically higher rate of platinum resistance than those in the stroma-low group; however, this difference did not reach statistical significance. These results should be interpreted with caution due to the retrospective study design and limited sample size; however, they nonetheless support further investigation of pretreatment TSR as a simple and easily applicable histopathological parameter in EOC. Larger prospective multicentre studies are required to determine whether this exploratory signal is reproducible and clinically relevant.

## Simple Summary

Platinum resistance remains an important challenge in the treatment of epithelial ovarian cancer (EOC). Identifying histopathological features associated with treatment response before therapy may help improve patient stratification. The tumour–stroma ratio (TSR) is a simple histopathological assessment method that can be applied on routine haematoxylin and eosin (H&E)-stained sections. In this study, stromal proportion was assessed using the TSR methodology and was evaluated on pretreatment diagnostic biopsy specimens in patients treated with neoadjuvant chemotherapy (NACT) followed by interval debulking surgery (IDS), and on primary surgical specimens in patients undergoing primary debulking surgery (PDS). Patients in the stroma-high group within the NACT + IDS cohort had a numerically higher rate of platinum resistance than those in the stroma-low group, although this difference was not statistically significant. Although these findings require confirmation in larger prospective multicentre studies, this exploratory observation supports further investigation of pretreatment stromal proportion in EOC.

## 1. Introduction

The development of platinum resistance represents a critical turning point in the clinical course of epithelial ovarian cancer (EOC), as it markedly limits therapeutic options and is strongly associated with adverse survival outcomes [1]. Accumulating evidence indicates that platinum resistance arises from a complex interplay of tumour-intrinsic alterations and extrinsic factors within the tumour microenvironment [2]. The tumour–stroma ratio (TSR) is a histopathological assessment method used to estimate the proportion of tumour-associated stroma within the tumour bed on routine haematoxylin–eosin sections [3]. In epithelial ovarian cancer, stromal predominance has been associated with advanced disease features and inferior survival outcomes, indicating that stromal proportion may reflect clinically relevant biological heterogeneity [4,5,6]. Across multiple solid malignancies, a stroma-rich phenotype has consistently been associated with adverse survival outcomes, and recent meta-analyses support the prognostic relevance of TSR assessment across different tumour types [7]. These findings suggest that histopathological assessment of stromal proportion may complement conventional clinicopathological parameters while remaining readily applicable in routine pathological assessment.

Although TSR has been investigated as a prognostic parameter in several solid tumours, its association with platinum resistance in epithelial ovarian cancer remains incompletely understood. In particular, the clinical relevance of stromal proportion assessed in pretreatment diagnostic biopsy specimens from patients receiving neoadjuvant chemotherapy followed by interval debulking surgery (NACT+IDS) remains poorly characterised. Therefore, the aim of this study was to evaluate the association of stromal proportion, assessed using the TSR methodology, with platinum resistance and survival outcomes in two separately analysed clinical cohorts: patients undergoing primary debulking surgery (PDS) and those receiving NACT followed by IDS, with particular emphasis on pretreatment stromal proportion assessed in diagnostic biopsy specimens from patients undergoing NACT followed by IDS.

## 2. Materials and Methods

### 2.1. Study Design and Patient Selection

This retrospective cohort study was performed at a tertiary referral centre in Izmir following approval by the institutional ethics committee (Approval No: 2025/176). Consecutive patients with histologically confirmed high-grade EOC who underwent cytoreductive surgery, either as primary debulking surgery (PDS) or after neoadjuvant chemotherapy (NACT) followed by interval debulking surgery (IDS), between January 2017 and January 2024 were screened for eligibility. Exclusion criteria comprised low-grade serous histology, the presence of another synchronous malignancy, diagnosis based solely on malignant ascites cytology without an available pretreatment tissue biopsy suitable for TSR assessment, and inadequate follow-up information. Demographic, clinicopathological, and treatment-related variables were retrieved from the institutional electronic medical database. The general clinical data collection procedures were consistent with those reported in a previously published study conducted at the same institution [8]. The present analysis was performed on the same patient population, with a distinct primary objective and an independent histopathological evaluation.

Patients analysed separately according to their initial treatment approach as the PDS and NACT+IDS cohorts. Within each cohort, subsequent analyses were performed according to stromal category (stroma-high vs. stroma-low).

### 2.2. Definitions and Endpoints

For the purposes of this retrospective analysis, platinum resistance was operationally defined as disease recurrence within six months after completion of first-line platinum-based chemotherapy, consistent with the conventional platinum-free interval-based definition used in historical ovarian cancer studies [9,10]. Postoperative residual disease was classified according to the maximal diameter of residual tumour as optimal (<1 cm) or suboptimal (≥1 cm) cytoreduction. The principal outcome measure was platinum resistance, whereas overall survival (OS) and disease-free survival (DFS) were analysed as secondary endpoints.

OS was calculated from the date of diagnosis to death from any cause, and DFS was calculated from the end of primary treatment to the first documented recurrence or death.

### 2.3. Assessment of the Tumour-Stroma Ratio (TSR)

Formalin-fixed, paraffin-embedded (FFPE) tissue specimens were retrieved from the institutional pathology archive. For patients who underwent primary debulking surgery (PDS), TSR was assessed on surgical resection specimens. For patients treated with neoadjuvant chemotherapy followed by interval debulking surgery (NACT+IDS), TSR was assessed exclusively on pretreatment diagnostic tissue biopsy specimens obtained before the initiation of systemic therapy. Given the different specimen types used for TSR assessment in the two treatment cohorts, direct comparisons of TSR distributions between the PDS and NACT+IDS groups were not performed. Patients diagnosed solely on the basis of malignant ascites cytology without an available pretreatment tissue biopsy suitable for TSR assessment were excluded from this study.

All available haematoxylin and eosin (H&E)-stained slides containing invasive tumour were reviewed for TSR assessment by a senior gynaecologic pathologist (D.A.), who was blinded to all clinicopathological characteristics, treatment strategy, platinum sensitivity status, and survival outcomes.

TSR assessment was performed according to previously published, internationally standardised recommendations for visual TSR evaluation on routine H&E-stained sections to ensure methodological consistency and reproducibility [11,12].

Initially, all tumour-containing slides were systematically screened at low magnification (×4–×5) to identify the most invasive viable tumour area. When multiple eligible slides or biopsy fragments were available, the specimen demonstrating the highest stromal proportion within the invasive tumour was selected for final assessment. The selected region was then examined at ×10 magnification. Only microscopic fields containing viable invasive tumour cells at all four borders of the field of view were considered suitable for scoring, ensuring that the evaluated stromal component represented tumour-associated stroma rather than adjacent non-neoplastic tissue. Stromal proportion was visually estimated in 10% increments [11,12].

The TSR assessment was based on the visually estimated proportion of tumour-associated stroma rather than on a mathematically calculated tumour-to-stroma quotient. Using the prespecified 50% cut-off, tumours with ≥50% stromal area were classified as stroma-high, whereas those with <50% stromal area were classified as stroma-low [11,12,13]. The terms “stroma-high” and “stroma-low” are used throughout the manuscript to distinguish the stromal proportion measured by the TSR methodology from a numerical tumour-to-stroma ratio.

Areas containing extensive necrosis, haemorrhage, mucin pools, tissue-processing artefacts, or normal ovarian tissue were excluded from evaluation. Small intratumoural blood vessels and tumour-associated inflammatory infiltrates were considered part of the stromal compartment, whereas large native blood vessels were avoided whenever possible [11,12].

**Figure 1:**
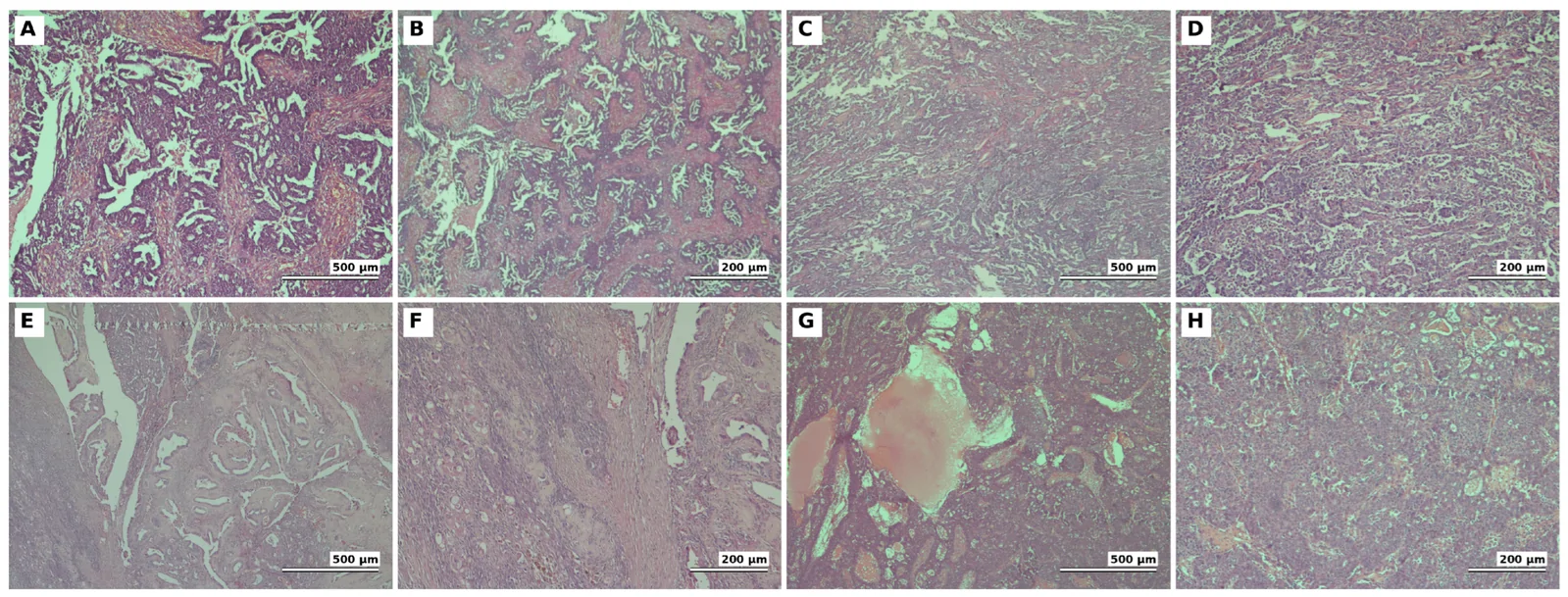
Representative haematoxylin and eosin (H&E)-stained histopathological sections illustrating tumour–stroma ratio (TSR) assessment in epithelial ovarian cancer. (**A**–**D**) Primary debulking surgery (PDS) cohort, serous histology: (**A**) stroma-low tumour at ×40 magnification; (**B**) stroma-low tumour at ×100 magnification; (**C**) stroma-high tumour at ×40 magnification; and (**D**) stroma-high tumour at ×100 magnification. (**E**–**H**) Neoadjuvant chemotherapy followed by interval debulking surgery (NACT+IDS) cohort, serous histology: (**E**) stroma-low tumour at ×40 magnification; (**F**) stroma-low tumour at ×100 magnification; (**G**) stroma-high tumour at ×40 magnification; and (**H**) stroma-high tumour at ×100 magnification. Scale bars: 500 µm for ×40 images and 200 µm for ×100 images.

Representative microphotographs illustrating stroma-low and stroma-high tumours are provided in Figure 1.

### 2.4. Statistical Analysis

Statistical analyses were performed using IBM SPSS Statistics (version 26.0) for Windows (IBM Corp., Armonk, NY, USA). Continuous variables were expressed as the mean ± standard deviation (SD) or median with interquartile range (IQR), as appropriate, and were compared using the independent samples t-test or the Mann–Whitney U test, depending on data distribution. Categorical variables were presented as frequencies and percentages and compared using the Chi-square test or Fisher’s exact test, as appropriate.

OS and DFS were estimated using the Kaplan–Meier method, and survival curves were compared using the log-rank test. OS was defined as the time from diagnosis to death from any cause, while DFS was defined as the time from completion of primary treatment to disease recurrence or death.

Owing to the limited number of events and the relatively small sample size, multivariable Cox proportional hazards regression analysis was not performed to avoid model overfitting and unstable estimates. As an exploratory analysis, univariable binary logistic regression was performed separately within the PDS and NACT+IDS cohorts to explore the association between stromal category (stroma-high vs. stroma-low) and platinum resistance. Platinum resistance was entered as the dependent variable, and stromal category was entered as the sole explanatory variable. Given the limited number of platinum-resistant events, particularly within the individual cohorts, multivariable logistic regression was not performed to avoid model overfitting and unstable effect estimates. Odds ratios (ORs) and corresponding 95% confidence intervals (CIs) were calculated.

A two-sided *p*-value of <0.05 was considered statistically significant.

## 3. Results

### 3.1. Patient Characteristics

A total of 83 patients with EOC were included in the analysis. The median age at diagnosis was 55 years (IQR: 45–61). The majority of patients (86.7%) had serous histology, and 67.5% were diagnosed at FIGO stage III–IV. All patients underwent debulking surgery, either as primary (66.3%) or interval (33.7%) procedures, with optimal debulking achieved in 81.9% of cases. Germline BRCA1/2 mutation status was available for 47 patients (56.7%), and 15 of them (18.1% of the cohort) tested positive.

Except for one patient who progressed during neoadjuvant treatment and one patient with stage IA disease who received only three cycles of adjuvant chemotherapy, all patients completed at least six cycles of platinum-based chemotherapy.

Baseline demographic and clinicopathological characteristics of the study population are summarised in Table 1.

In the PDS cohort, platinum resistance did not differ according to TSR status (0% vs. 16.7%, Fisher’s exact *p* = 0.577). In the NACT+IDS cohort, platinum resistance was numerically more frequent in the stroma-high group than in the stroma-low group (57.9% vs. 22.2%), although the difference did not reach statistical significance (Fisher’s exact *p* = 0.114).

### 3.2. Clinicopathological Characteristics According to Stromal Category

Within the PDS cohort, no significant differences in baseline clinicopathological characteristics were observed between the stroma-low and stroma-high groups. In the PDS group, mean age did not differ significantly between patients in the stroma-low and stroma-high groups (52.71 ± 13.03 vs. 53.31 ± 8.99 years; *p* = 0.877). Histological subtype distribution and FIGO stage were also comparable between stromal categories (*p* = 0.134 and *p* = 1.000, respectively). Similarly, germline BRCA mutation status, residual disease status, platinum resistance and recurrence rates did not differ significantly according to stromal category in patients undergoing PDS (all *p* > 0.05).

In the NACT+IDS cohort, patients in the stroma-high group showed a numerically higher rate of platinum resistance than those in the stroma-low group (57.9% vs. 22.2%). However, this difference did not reach statistical significance (Fisher’s exact *p* = 0.114). Age, BMI, baseline CA-125 level, germline BRCA mutation status, residual disease status, and recurrence rates were also comparable between stromal categories (all *p* > 0.05). Given the limited number of platinum-resistant events, no multivariable logistic regression analysis was performed.

Overall, stromal category was not significantly associated with the evaluated clinicopathological characteristics in either cohort. The numerical difference in platinum resistance observed between the stroma-high and stroma-low groups in the NACT+IDS cohort did not reach statistical significance.

### 3.3. Exploratory Univariable Logistic Regression Analysis for Platinum Resistance

To further explore the association between stromal category and platinum resistance, univariable binary logistic regression analyses were performed separately within the PDS and NACT+IDS cohorts. The PDS cohort included 55 patients and 8 platinum-resistant events; however, the regression estimate was unstable because no platinum-resistant events occurred in the stroma-low group and was therefore not considered clinically interpretable. In the NACT+IDS cohort, which included 28 patients and 13 platinum-resistant events, the stroma-high group had higher estimated odds of platinum resistance than the stroma-low group (OR 4.81, 95% CI 0.78–29.59; *p* = 0.090). This finding did not reach statistical significance, and the wide confidence interval indicates substantial uncertainty in the effect estimate (Table 2).

### 3.4. Survival Analysis

OS and DFS were analysed using the Kaplan–Meier method and compared according to stromal category within the PDS and NACT+IDS cohorts.

In the PDS cohort, no significant difference in DFS was observed between patients in the stroma-low and stroma-high groups (log-rank *p* = 0.614). Similarly, OS did not differ significantly according to stromal category in this group (log-rank *p* = 0.330).

In the NACT+IDS cohort, DFS was comparable between the stroma-low and stroma-high groups (log-rank *p* = 0.445). Likewise, no statistically significant difference in OS was detected according to stromal category (log-rank *p* = 0.228).

**Figure 2:**
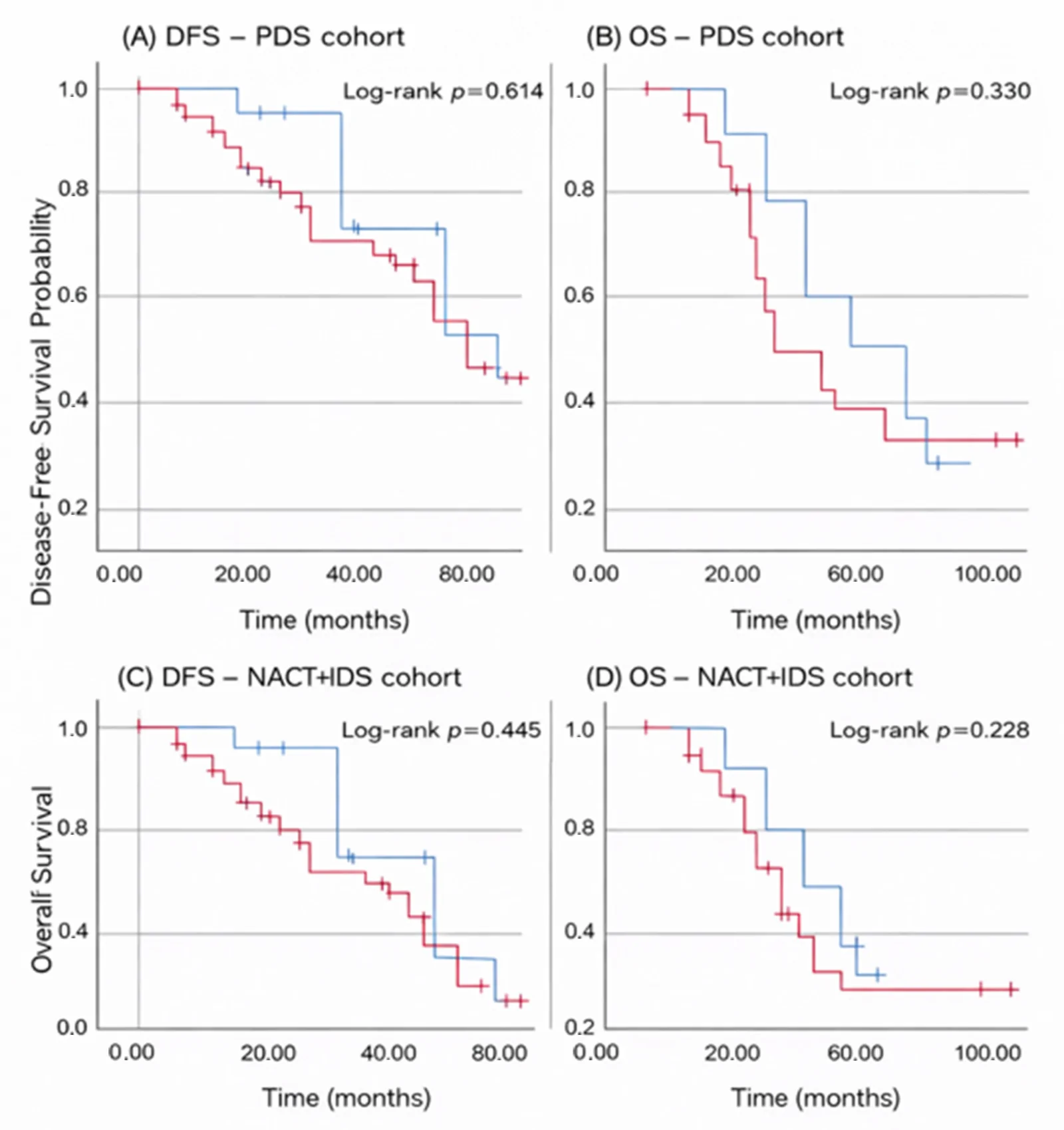
Stromal category and survival outcomes. Kaplan–Meier survival curves for disease-free survival (DFS) and overall survival (OS) stratified by stromal category. (**A**) DFS and (**B**) OS in the primary debulking surgery (PDS) cohort; (**C**) DFS and (**D**) OS in the neoadjuvant chemotherapy followed by interval debulking surgery (NACT+IDS) cohort. Patients were grouped as stroma-low (<50% stromal area) or stroma-high (≥50% stromal area). The blue curves represent the stroma-low group, whereas the red curves represent the stroma-high group. Tick marks represent censored observations. Log-rank *p*-values are shown.

Kaplan–Meier survival curves for DFS and OS stratified by stromal category in both cohorts are presented in Figure 2. No statistically significant association between stromal category and either DFS or OS was observed in the PDS or NACT+IDS cohort.

## 4. Discussion

The aim of this study was to evaluate the clinical significance of stromal proportion, assessed using the TSR methodology, in relation to platinum resistance and survival outcomes in patients with EOC analysed separately within the PDS and NACT+IDS cohorts. In the present study, stromal proportion in the NACT+IDS cohort was assessed on pretreatment diagnostic biopsy specimens obtained before the initiation of neoadjuvant chemotherapy, whereas stromal proportion in the PDS cohort was evaluated on primary surgical resection specimens. Our findings showed a numerically higher rate of platinum resistance among patients in the stroma-high group in the NACT+IDS cohort than among those in the stroma-low group; however, this difference did not reach statistical significance. No significant association was identified between stromal category and survival outcomes in either cohort. These findings support further investigation of TSR according to treatment strategy in larger prospective multicentre studies.

Previous studies have demonstrated an association between increased tumour–stroma proportion and adverse clinical outcomes in EOC. Lou et al. first reported an association between high tumour–stroma proportion and platinum resistance, which was subsequently confirmed in a larger independent cohort, where high tumour–stroma proportion was also associated with shorter PFS and OS [13,14]. More recently, Collet et al. demonstrated the prognostic value of tumour–stroma proportion in pretreatment tumour samples from patients with advanced EOC receiving neoadjuvant chemo-immunotherapy [15]. In our study, stromal proportion in the NACT+IDS cohort was assessed using pretreatment diagnostic biopsy specimens obtained before NACT, allowing assessment of the baseline tumour microenvironment before treatment exposure. Although pretreatment stromal proportion was not significantly associated with platinum resistance, patients with high pretreatment TSR showed a numerically higher rate of platinum resistance than those in the stroma-high group. This numerical trend warrants further investigation in larger, adequately powered cohorts but does not establish pretreatment stromal proportion as a marker of platinum response.

The biological mechanisms linking stromal abundance to platinum resistance remain incompletely understood. Increasing evidence suggests that the tumour stroma is not merely a passive structural component but an active regulator of tumour behaviour and treatment response. Cancer-associated fibroblasts (CAFs), one of the major constituents of the tumour stroma, have been shown to promote tumour progression and contribute to platinum resistance through paracrine signalling, extracellular matrix remodelling, activation of multiple oncogenic pathways—including TGF-β, PI3K/AKT, and MAPK—and modulation of the immune microenvironment [16,17,18,19,20]. In addition, the stromal compartment influences the composition and function of the immune microenvironment, and increased tumour-infiltrating lymphocytes (TILs) have been associated with improved response to platinum-based chemotherapy and favourable survival outcomes in EOC [21,22,23]. Patients selected for NACT+IDS generally represent a population in which complete primary cytoreduction is considered unlikely because of disease distribution and/or tumour burden [24]. Accordingly, a stroma-high phenotype in pretreatment TSR biopsy specimens may reflect differences in the underlying tumour microenvironment and disease biology that may contribute to the numerically higher rate of platinum resistance observed in the NACT+IDS cohort [25,26]. However, given the absence of statistical significance and the limited sample size, this biological interpretation remains hypothetical and requires validation in larger prospective cohorts.

The absence of a similar pattern in the PDS cohort should be interpreted with caution. Patients undergoing PDS and those treated with NACT+IDS represent clinically distinct populations. According to current ASCO guidelines, NACT is generally recommended for patients in whom complete primary cytoreduction is considered unlikely because of disease distribution and/or tumour burden, whereas PDS remains the preferred approach when complete cytoreduction is considered feasible [24,27]. Importantly, stromal proportion was assessed using different specimen types in the two cohorts: primary surgical resection specimens in the PDS cohort and pretreatment diagnostic biopsies in the NACT+IDS cohort. Therefore, the differences observed between the cohorts cannot be attributed to treatment strategy itself and may reflect differences in specimen type and sampling, as well as baseline disease characteristics, patient selection, and underlying tumour biology [8,28]. Prospective studies using standardised tissue sampling and stromal assessment are needed to enable valid cross-cohort comparisons.

This study has several strengths worth noting. In particular, stromal proportion in the NACT+IDS cohort was assessed using pretreatment diagnostic biopsy samples, which allowed for the evaluation of the baseline tumour microenvironment prior to treatment exposure. Furthermore, the separate analysis of the PDS and NACT+IDS cohorts made it possible to evaluate stromal proportion within two clinically distinct cohorts while avoiding direct cross-cohort comparisons. However, there are also several limitations to consider. First, this was a retrospective, single-centre study with a relatively small sample size, particularly in the NACT+IDS cohort, which included 28 patients and 13 platinum-resistant events. This limited sample size reduced the statistical precision of the subgroup analysis, as reflected by the wide confidence interval around the effect estimate. Accordingly, multivariable logistic regression was not performed, and the observed numerical difference in platinum resistance should be regarded as an exploratory, hypothesis-generating signal rather than evidence of an independent or predictive association. Second, in the NACT+IDS cohort, stromal proportion was assessed on pretreatment diagnostic biopsy specimens, which may not fully capture the spatial heterogeneity of the tumour microenvironment across the entire tumour burden [28]. Although all available tumour-containing biopsy material was reviewed, sampling from a limited tissue area remains an inherent limitation of biopsy-based assessment. Future studies incorporating multi-region sampling and automated digital pathology approaches may enable more comprehensive and reproducible quantification of stromal features and help characterise intratumoral spatial heterogeneity [29,30,31]. Third, stromal proportion was assessed using different specimen types in the two cohorts, namely surgical resection specimens in the PDS cohort and pretreatment diagnostic biopsies in the NACT+IDS cohort. This methodological difference precludes direct comparison of stromal findings between the cohorts and limits any inference regarding treatment strategy itself.

Fourth, platinum resistance was defined using the conventional six-month platinum-free interval threshold. Although this definition facilitates comparison with historical studies, contemporary management of recurrent EOC increasingly recognizes platinum sensitivity as a continuum influenced by multiple clinical factors rather than as a strictly dichotomous classification [9,10]. Accordingly, our operational definition may not capture the full complexity of treatment responsiveness in current clinical practice.

Finally, the lack of an external validation cohort with comparable pretreatment biopsy material, treatment information, and platinum resistance outcomes limits the generalisability of our findings. Therefore, larger prospective multicentre studies with standardised pretreatment tissue sampling and independent validation are needed to determine whether the exploratory signal observed in the NACT+IDS cohort is reproducible and clinically relevant.

## 5. Conclusions

In conclusion, patients in the stroma-high group in the NACT+IDS cohort showed a numerically higher rate of platinum resistance than those in the stroma-low group; however, this difference did not reach statistical significance. This exploratory finding should be interpreted with caution given the retrospective design and limited sample size of this study. No significant association between stromal category and survival outcomes was observed in either cohort. Larger prospective multicentre studies using standardised pretreatment tissue assessment are required to determine whether the exploratory signal observed in the NACT+IDS cohort is reproducible and clinically relevant.

## Figures and Tables

**Table 1:** Clinicopathological characteristics according to stromal category within the PDS and NACT+IDS cohorts.

	All (*n* = 83)	PDS			NACT+IDS		
		Stroma-Low (*n* = 7)	Stroma-High (*n* = 48)	*p* *	Stroma-Low (*n* = 9)	Stroma-High (*n* = 19)	*p* *
Age (years) Mean ± SD	54.24 ± 10.30	52.71 ± 13.03	53.31 ± 8.99	0.877	53.88 ± 12.62	57.31 ± 11.47	0.481
Histology							
Serous	72 (86.7%)	4 (57.1%)	40 (83.3%)	0.134	9 (100%)	19 (100%)	
Others	11 (13.3%)	3 (42.9%)	8 (16.7%)				
Stage (FIGO)							
I–II	27 (32.5%)	3 (42.9%)	24 (50%)	1.000	0 (0%)	0 (0%)	
III–IV	56 (67.5%)	4 (57.1%)	24 (50%)		9 (100%)	19 (100%)	
BMI (kg/m^2^), mean ± SD	24.63 ± 1.33	24.47 ± 1.05	24.66 ± 1.49	0.739	24.42 ± 1.22	24.67 ± 1.08	0.588
Baseline CA-125 (U/mL), mean ± SD	827.35 ± 1397.42	401 ± 271.97	429.66 ± 530.25	0.757	1467.77 ± 892.21	1685.73 ± 2511.6	0.595
gBRCA mutation †							
Negative	32 (38.6%)	2 (28.6%)	16 (33.3%)	0.844	4 (44.4%)	10 (52.6%)	0.127
Positive	15 (18.1%)	1 (14.3%)	10 (20.8%)		5 (55.6%)	9 (47.4%)	
Residual Disease							
Optimal (<1 cm)	68 (81.9%)	7 (100%)	38 (79.1%)	0.328	9 (100%)	14 (73.7%)	0.144
Suboptimal (≥1 cm)	15 (18.1%)	0	10 (20.8%)		0	5 (26.3%)	
Platinum Resistance ‡							
No	62 (74.7%)	7 (100%)	40 (83.3%)	0.577	7 (77.8%)	8 (42.1%)	0.114
Yes	21 (25.3%)	0	8 (16.7%)		2 (22.2%)	11 (57.9%)	
Recurrence							
No	36 (43.4%)	5 (71.4%)	27 (56.3%)	0.686	1 (11.1%)	3 (15.8%)	1.000
Yes	47 (56.6%)	2 (28.6%)	21 (43.7%)		8 (88.9%)	16 (84.2%)	

Values are presented as mean ± standard deviation or number (%), as appropriate. *p* * values were calculated using the Chi-square test, Fisher’s exact test, Student’s *t*-test, or Mann–Whitney U test, as appropriate. Other histological subtypes included clear cell carcinoma (n = 7) and endometrioid carcinoma (n = 4). † Germline BRCA mutation status was available for 47 patients. ‡ Platinum resistance was defined as disease recurrence within six months after completion of first-line platinum-based chemotherapy. Abbreviations: PDS, primary debulking surgery; NACT, neoadjuvant chemotherapy; IDS, interval debulking surgery.

**Table 2:** Exploratory univariable logistic regression analysis for platinum resistance in the NACT+IDS cohort.

Variable	OR	95% CI	*p*-Value
Stroma-high vs. stroma-low	4.81	0.78–29.4	0.090

Univariable binary logistic regression with platinum resistance as the dependent variable and stromal category (stroma-high vs. stroma-low) as the sole explanatory variable. OR, odds ratio; CI, confidence interval.

## Data Availability

The datasets generated and/or analysed during the current study are available from the corresponding author upon reasonable request.

## Abbreviations

The following abbreviations are used in this manuscript:

CAFs	Cancer-associated fibroblasts
CI	Confidence interval
DFS	Disease-free survival
EOC	Epithelial ovarian cancer
FIGO	International Federation of Gynecology and Obstetrics
gBRCA	Germline BRCA1/2 mutation
H&E	Haematoxylin and eosin
IDS	Interval debulking surgery
IQR	Interquartile range
NACT	Neoadjuvant chemotherapy
OR	Odds ratio
OS	Overall survival
PDS	Primary debulking surgery
SD	Standard deviation
TSR	Tumour–stroma ratio
